# Influence of Beam Figure on Porosity of Electron Beam Welded Thin-Walled Aluminum Plates

**DOI:** 10.3390/ma15103519

**Published:** 2022-05-13

**Authors:** Matthias Moschinger, Florian Mittermayr, Norbert Enzinger

**Affiliations:** 1 Institute of Materials Science, Joining and Forming, Graz University of Technology, 8010 Graz, Austria; norbert.enzinger@tugraz.at; 2 Institute of Technology and Testing of Building Materials, Graz University of Technology, 8010 Graz, Austria; f.mittermayr@tugraz.at

**Keywords:** electron beam welding, aluminum 6082, porosity, beam figure

## Abstract

Welded aluminum components in the aerospace industry are subject to more stringent safety regulations than in other industries. Electron beam welding as a highly precise process fulfills this requirement. The welding of aluminum poses a challenge due to its high tendency to pore formation. To gain a better understanding of pore formation during the process, 1.5 mm thick aluminum AW6082 plates were welded using specially devised beam figures in different configurations. The obtained welds were examined with radiographic testing to evaluate the size, distribution, and the number of pores. Cross-sections of the welds were investigated with light microscopy and an electron probe microanalyzer to decipher the potential mechanisms that led to porosity. The examined welds showed that the porosity is influenced in various ways by the used figures, but it cannot be completely avoided. Chemical and microstructural analyzes have revealed that the main mechanism for pore formation was the evaporation of the alloying elements Mg and Zn. This study demonstrates that the number of pores can be reduced and their size can be minimized using a proper beam figure and energy distribution.

## 1. Introduction

Saving fuel and increasing payload are two of the biggest goals in aviation and aerospace. Manufacturing parts in lightweight construction is one way to support this goal. Low density and high strength make aluminum an excellent construction material for such lightweight components that are already being used in this sector. However, the joining of aluminum materials is often challenging [1,2]. Especially in the aerospace industry, the limitations regarding defect sizes and processes are obviously very strict [3].

Porosity in aluminum welding is a well-known and critical phenomenon. According to the literature, the formation of porosity in the weld can have different reasons, such as the processes, but also the base materials’ chemical composition and/or filler materials play an important role. For instance, the introduction of hydrogen into the molten pool is of great importance, as it is highly soluble in liquid aluminum [4].

The alloy burn-off of certain elements is a further mechanism causing pore formation. Zhang et al. described the effect on electron beam welded (EBW) AA6062 [5]. Zhou et al. investigated this elemental burn-off effect in detail in their article on plasma welding of an AW5052 [6]. The welded Al-alloys in both of these studies displayed the burn-off mechanism for Mg. 

The formation of a gaseous phase from aluminum and its oxide is another pore-forming mechanism that is related to the chemistry of the material as described by Fujii et al. [7]. According to the authors, it can only occur in a high vacuum, such as during the EBW process due to the low partial pressure of the oxide. Nogi et al. [8] showed that the oxide thickness correlated with the porosity during EBW. This is also a conceivable mechanism for porosity, but it is difficult to detect. 

There is also the possibility of pore formation solely from the process side. During EBW, a keyhole can form due to the high energy density, which causes complex flow processes in the weld pool [9]. However, these processes have not yet been fully understood as the experimental detection of such flows is a challenge [10,11]. Chen et al. suggested that these flows cause the movement of bubbles within the melt pool. If the bubbles come close to the solidification front, they can be “frozen” and thus remain as pores in the weld. 

The beam deflection also leads to a manipulation of the molten pool and thus influences the fluid flow. At present, the influences on the melt pool of beam oscillation are rarely investigated. Wang et al. [12] studied oscillation movements describing the behavior of longitudinal, transverse, and circular oscillation on the molten bath and its influence on solidification patterns. In the case of superimposed oscillations, the shape of the “eight” appears in the literature, in addition to the circle; both represent so-called Lissajous figures. Kabasakaloglu et al. [13] and Chen et al. [14] investigated the influences of the beam deflection on the porosity. In both studies the figure of the “eight”, either standing (8) or lying (∞) was selected.

The latter mentioned studies were all performed on laser welded aluminum under atmospheric conditions. Since the laser beam is always limited in its deflection speed (inertia due to robot mass, mirror kinematics), only beam deflections in the lower speed spectrum can be achieved. On the contrary, in the case of EBW, the electron beam is moved using magnetic fields, and thus a significantly higher deflection speed can be realized. Thus, the current study deals with the influences of electron beam deflection on porosity. For this purpose, new beam figures were devised for the welding process. The aims of this study are (i) to counteract the formation of gas bubbles by optimizing the beam figures, (ii) to facilitate the escape of potential bubbles from the melt pool by extending it, and (iii) to investigate the underlying mechanisms that lead to porosity during EBW on AW6082. 

## 2. Materials and Methods

### 2.1. Material

The aluminum used in this work is the commercial alloy AW6082-T6, i.e., in the aged condition [15]. The chemical limits of the composition of the material used are shown in Table 1. The used specimens were plates with a length of 300 mm and a width of 60 mm. The plates have a thickness of 1.5 mm, but they have a material accumulation at their butt edge to ensure sufficient material supply during the welding process. This is necessary due to the required seam elevation and root penetration. The joint geometry is produced by milling. This processing also has the purpose of removing the existing oxide layer. To prevent a renewed formation of this oxide layer as far as possible, the milled plates were packed airtight directly after processing.

### 2.2. Welding Process and Beam Figures

The EBW machine used was a chamber system of the type EBG 45-150K14 from Pro-beam GmbH & Co. KGaA. Since EBW allows a wide range of welding parameter combinations, welding parameters were determined by preliminary tests. For this purpose, blind welds were first welded and the parameters obtained were adapted to the joint geometry. The majority of the weld parameters were kept constant over all tests. The EB was focused on the plate surface. Welding was performed at a welding speed of 20 mm/s. The chamber pressure during welding was below 5 × 10^−4^ mbar. The beam oscillation was achieved using beam figures with a frequency of 500 Hz. Welding was performed with a beam voltage of 80 kV. The beam power could not be kept constant over all tests, because the beam figures had different shapes and different energy distributions. Therefore, the beam current varied in the range of 9.7–13 mA.

(1) $$t\;\to \left(\begin{array}{c} A_{x}\sin\left(\omega_{1}t+\varphi_{1}\right)\\ A_{y}\sin\left(\omega_{2}t+\varphi_{2}\right) \end{array}\right),\;\;t\in \left[0,\infty \right)$$

**Figure 1:**
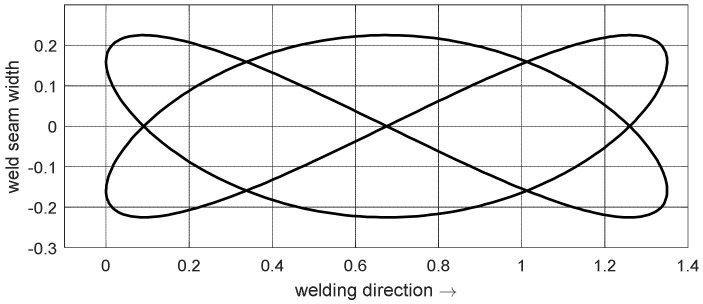
Reference figure R which is the basis for the devised figures.

**Figure 2:**
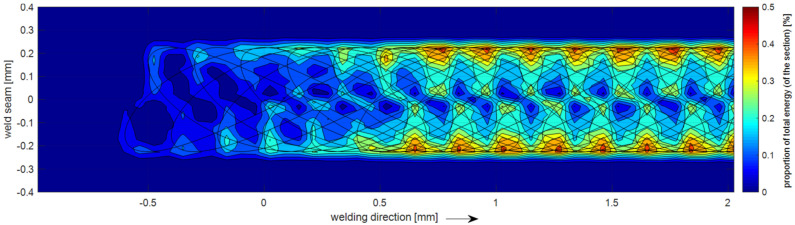
Relative energy distribution of the moving reference figure R starting on the left and reaching steady state on the right side.

The beam oscillated using superimposed oscillations in x and y-directions which were parameterized according to Equation (1). A frequency ratio *ω_1_*:*ω*_2_ of 2:3 and a phase shift of Δ$\varphi =\varphi_{1}-\varphi_{2}=0{}^{\circ}$ resulted in a figure as shown in Figure 1. This Lissajous figure represents the reference figure *R* with an overall width of 0.45 mm and a total length of 1.35 mm. All other figures were derived from the reference figure: Several variations of the figure were created which differ in terms of their energy distribution and dimensions. To evaluate the overall energy distribution, the trajectory of the figure was analyzed, as shown in Figure 2. For this purpose, the points describing the beam figure were considered to have a constant energy input and were integrated over the welding path.

**Figure A1:**
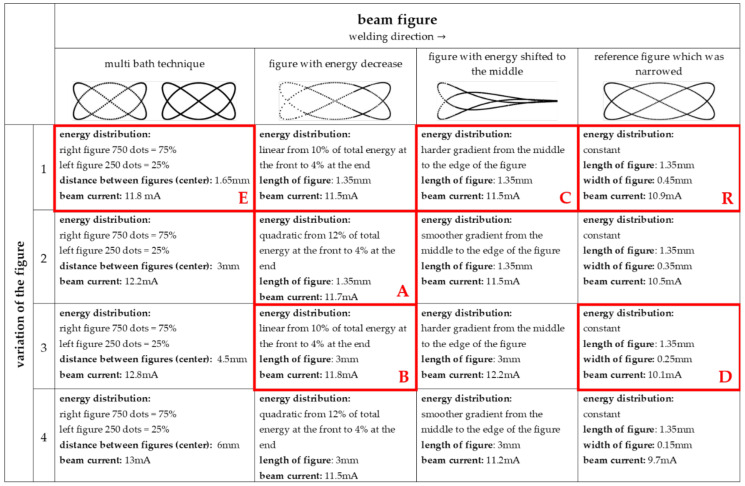
Test matrix from the first set of experiments. Marked in red are the six chosen figure configurations and their parameters which were applied in the second set of experiments.

In total 46 welding experiments were performed, 16 in the first set and 30 in the second set of experiments. In the first set, four figures were welded in four different configurations (the configurations are shown in Figure A1 in Appendix A). The resulting 16 welds were examined and based on these preliminary results a second experimental set was designed and consequently welded (marked in red and labeled in Table A1).

For this second experiment set, five different figure configurations were selected from the first experiment set (see Table 2 and Table A1). At least one configuration of each figure was repeated, the decision was based on porosity and weld appearance (root and seam). The figure configurations of series A-C & E were chosen for their low porosity appearance. Figure configuration D was chosen because of the high pore count. It was investigated whether high porosity can be reproduced. The set was completed by the reference figure R. Every configuration was repeated five times, to investigate the reproducibility of the results. This resulted in a total weld length of 1.3 m minus the start and end sections of the welded plates, so only the stationary part of the weld was investigated.

- The series A and B were derived from the same basic figure which has an energy reduction from the front to the back, which was either linear or quadratic. In addition to the energy distribution, the length of the figure was also changed. Thus, A and B represent two figure configurations with different lengths and energy distributions as shown in Table 2.
- For the basic figure of the series C, the energy was shifted to the weld centerline, since the analysis of the energy distribution of the reference figure R showed that most of the energy accumulated on the figure edge (see Figure 2). The figure was varied in length and the gradient of the energy distribution, the series C configuration was finally chosen for this figure type.
- Series D was developed based on the reference figure R with constant energy distribution. However, the width was decreased to make the weld narrower and thus give the pores less space to form. The selected figure width for series D was 0.25 mm (see Figure 2).
- The basis for the series E was a multi-bead technique as known from the literature [17]. Here, two figures were applied sequentially, the first figure ensuring the full penetration welding; the second figure is a post-heating of the molten pool. The distance between the individual figures (center to center) of 1.65 mm was used, which leads to a total length of 3 mm (front of the right figure to back of the left figure).

**Figure 3:**
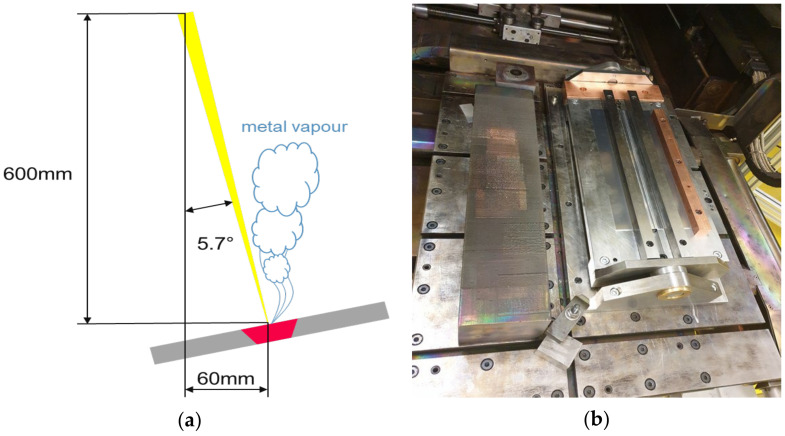
(**a**) Schematic of the tilted beam; (**b**) Pivoting clamping device with welded plates.

Due to the low beam current (9.7–13 mA) of the EB and thus the low beam power the EB was susceptible to disturbances. Such a disturbance can be, e.g., the rising metal vapor during the process. To counteract metal vapor disturbances, the beam was tilted by 5.7°, see Figure 3a. The tilting of the EB distorted the beam figure in the horizontal plane. To correct this, the plates were also tilted at this angle using a swiveling clamping device (Figure 3b).

Before mounting, the plates were unpacked and cleaned with isopropanol. To minimize oxidation as much as possible, the welding chamber was closed and evacuated about 3 min after unpacking the sample.

Before actual welding, the plates were tack welded using the reference figure R with a current of 6 mA. The tack weld was welded over a length of 290 mm, of which 15 mm was each slope in and slope out. This slope in/out was not considered for the evaluation, and therefore, arbitrarily selected; it was intended to provide a smooth entry and exit area for the weld. After tack welding, a waiting time of 5 min was considered before the actual welding process was carried out, also with the slope in/out. After welding, a 2 min waiting time was defined before the chamber was flooded with air, and the plates were removed from the machine.

### 2.3. Investigations

From the first experimental set, samples were taken 50 mm from the beginning of the weld and analyzed metallographically. The longer parts of the weld, about 220 mm each, were examined for pores using radiographic testing. The second experimental set was only investigated radiographically.

To investigate the microstructure, cross-sections were prepared using BARKER etching [18]. The etching was carried out at 25 V with a flow rate of 15 L/min over a time of 120 s. A light microscope (Zeiss Axio Observer Inverted) was used to view the cross-sections. Images were taken using polarized light incident at an angle of 3.5° on the sample surface.

**Figure 4:**
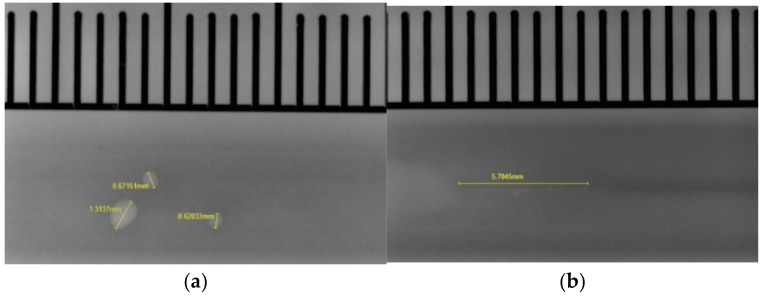
(**a**) example of an X-ray image of the test series D with different sized pores; (**b**) misinterpreted weld butt at the end of the weld.

To detect the porosity in the welds, radiographic testing was used. The entire length of the weld was irradiated without further sample preparation. This was conducted with the Yxlon Cheetah system, which was operated with a microfocus X-ray tube. The working voltage of the tube was 120 kV at 90 mA current. The radiography resulted in 15 X-ray images covering the entire length of the weld. An example image is shown in Figure 4a. First, the images were automatically analyzed using the Yxlon Fgui software with 14× magnification. Then the X-ray images were manually re-inspected and the data were corrected as the software also identified non-porosity related objects (e.g., weld butts) in the areas of weld start/end see Figure 4b.

The chemical composition combined with the microstructure of welds and the base material was analyzed using an EPMA (Electron Probe Microanalyzer, JEOL JXA8530F Plus Hyper Probe) equipped with a Schottky field emission gun on the welded reference sample. Therefore, several cross-sections through welds (with and without visible pores) were pre-selected from the light microscopic investigations, polished and C-coated. Images were recorded in the backscattered electron (BSE) and in the secondary electron (SE) modes at 15 kV acceleration voltage and 20 nA beam current. Semi-quantitative elemental distribution images of aluminum, magnesium, silicon, manganese and iron were acquired from selected areas with the same acceleration voltage, a beam current of 50 nA, a dwell time of 15 mS and a focused beam. The dimension of the mapping showing both sides of the fusion line (Figure 7 and Figure A3) is 1000 × 350 px and was recorded with a 1µm step size, while mappings with a large pore (Figure 8 and Figure A4) are 1000 × 1000 px and were recorded with a 0.5 µm step size. The quantification of the individual element mappings in wt.% was performed against certified pure metal standards. The quantitative single spot analyses were performed on the Al-phase both in the welds and base material. The Si-eutectic and other phases were not investigated. The analytical conditions were as follows: 15 kV accelerating voltage, 20 nA beam current and a defocused beam with a diameter of 40 μm. The full quantitative chemical analyses were also standardized against certified pure metal standards and included the following elements: Al, Si, Mg, Fe, Cu, Zn, Mn and Cr. The counting times on peak and on both sides on the background positions were 20 and 10 s, respectively. The detection limits were 117 ppm for Al, 89 ppm for Si, 140 ppm for Mg, 238 ppm for Fe, 294 ppm for Cu, 639 ppm for Zn, 216 ppm for Mn and 246 ppm for Cr. Results presented in Table 3 show integrated averages in wt.% from the highlighted areas in Figure 7 and Figure A4. For the single spot analyses, mean values for the weld areas (*n* = 27) and the base material (*n* = 18) are shown. The results of the individual single spot analyses are placed in the Appendix (Table A1).

## 3. Results

### 3.1. Metallography

**Figure 5:**
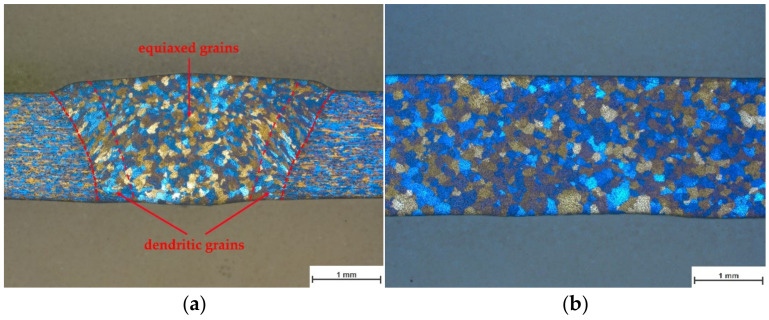
(**a**) cross-section of the reference figure R; (**b**) longitudinal section of the reference figure.

**Figure A2:**
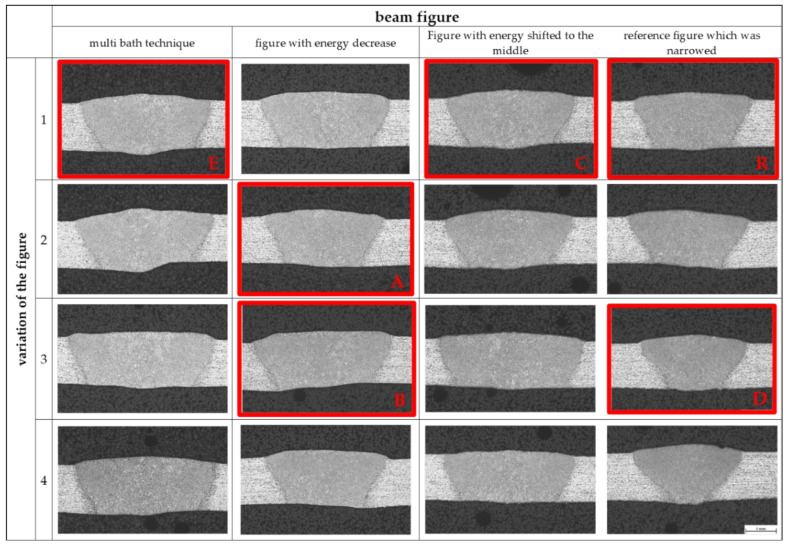
Cross-sections from the first set of experiments. The microstructures outlined in red are the representatives for the second set of experiments and marked like this.

**Figure A3:**
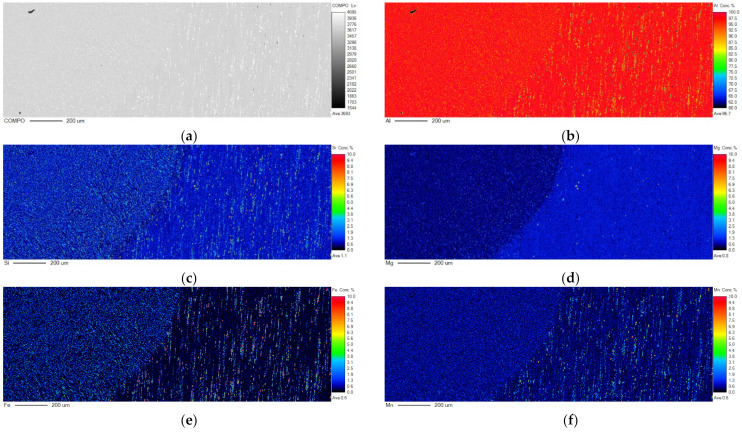
(**a**) BSE image and elemental distribution mappings: (**b**) Al, (**c**) Si, (**d**) Mg, (**e**) Fe, (**f**) Mn.

**Figure A4:**
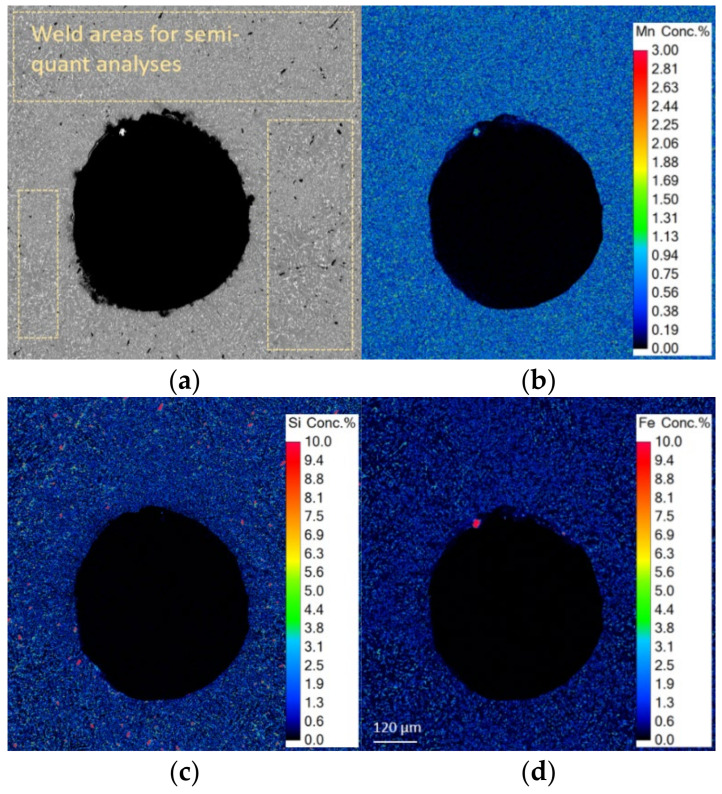
(**a**) BSE image showing a large pore (Ø≈0.7 mm) in a weld. The integrated area of all three indicated yellow rectangles were used to semi-quantitively evaluated the elemental mappings for Al, Mg (both shown in Figure 8), (**b**) Mn, (**c**) Si and (**d**) Fe.

The cross-sections showed little difference in shape and microstructure (Figure A2). In Figure 5a, the cross-section of reference R is shown. The microstructural investigation valid for all welding tests clearly revealed two different areas, a dendritic area starting from the fusion line and a globular area in the center of the weld. The globular region represents equiaxed grains, as shown by a longitudinal section in the middle of the weld (Figure 5b).

### 3.2. Porosity

**Figure 6:**
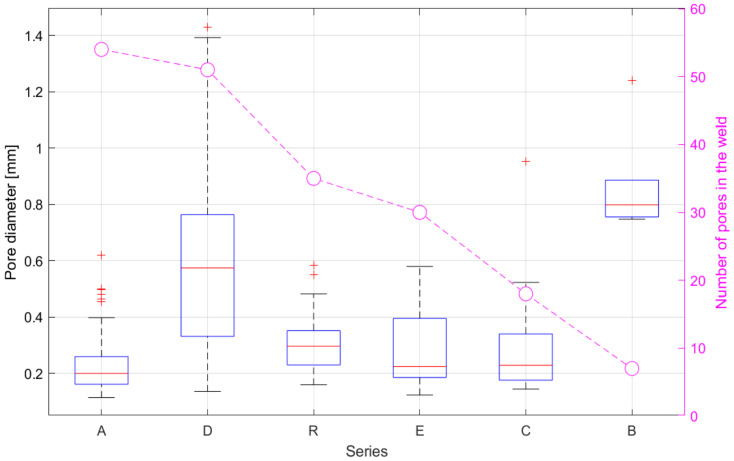
Number of pores and their pore diameter distribution from the second set of experiments.

The results shown in Figure 6 are from the second set of experiments. The diagram shows, on the one hand, the number of pores and on the other hand a boxplot diagram [19] of the pore diameters. It can be seen that there are significant differences between the tests, in terms of the number of pores, and the average pore diameter. For instance, series B has the lowest number of pores (7), but the largest average pore diameter (0.80 mm). On the contrary, series A has the most pores (54), but the smallest average pore diameter (0.20 mm) and a low deviation around the average pore size. Series D recorded a high number of pores (52), the second largest average pore size (0.58 mm) of all tests, and by far the largest deviation around the average. Series C emerged with a relatively low number of pores (18), while also having a relatively low average pore diameter of 0.22 mm. The reference test R and series E had similar numbers of pores (R: 35; E: 30). However, these two tests had slightly different average pore sizes of 0.30 and 0.24 mm. 

### 3.3. Chemical Composition and Microstructure Revealed by EPMA

**Figure 7:**
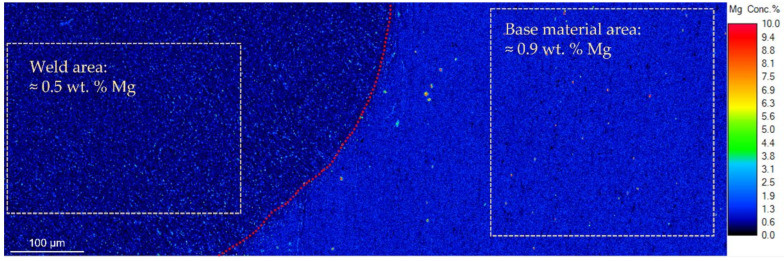
Detection of magnesium in the weld (**left**) via the fusion line (red dotted line) into the base material (**right**).

The following investigations were performed on samples that were welded with the reference beam figure R. The intense heating as a consequence of the EBW process clearly caused (micro)structural and chemical modifications to the Al-alloy. Figure 7, for example, shows an elemental mapping of magnesium. The fusion line (indicated in red) is well visible and it marks a distinct structural and chemical border between the base material (right side) and the weld area (the left side of the image is the center of the weld). The Mg distribution shows a more homogeneous distribution and the presence of relatively large Mg and Si containing phases in the base material, which is due to the aging process of the alloy. On the contrary, in the weld, the Mg is depleted and much less homogeneously distributed. The quantification of the map and the results from the single spot analyses (see Table 3 and Table A1) both indicate that the resolidified weld contained approximately 40 wt.% less Mg compared to the base material. For all the other elements, the analyses of the base metal match well with the boundary values given for AW6082 (see Table 1). Besides the Mg depletion, a clear reduction of Zn was recorded. In the base material 0.13 ± 0.02 wt.% Zn was found while in the weld the Zn-content was below the detection level of ≈600 ppm.

**Figure 8:**
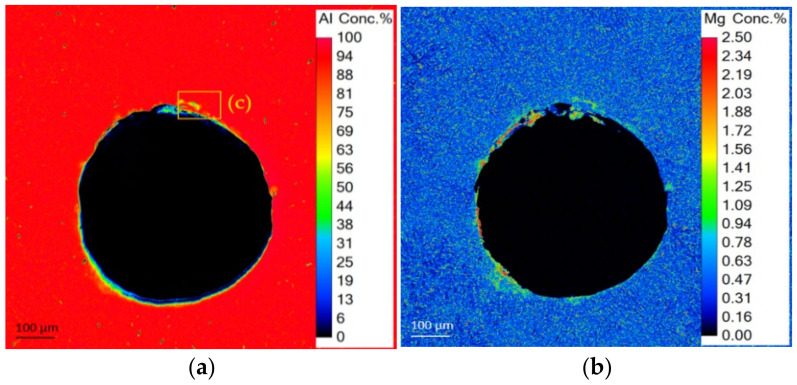

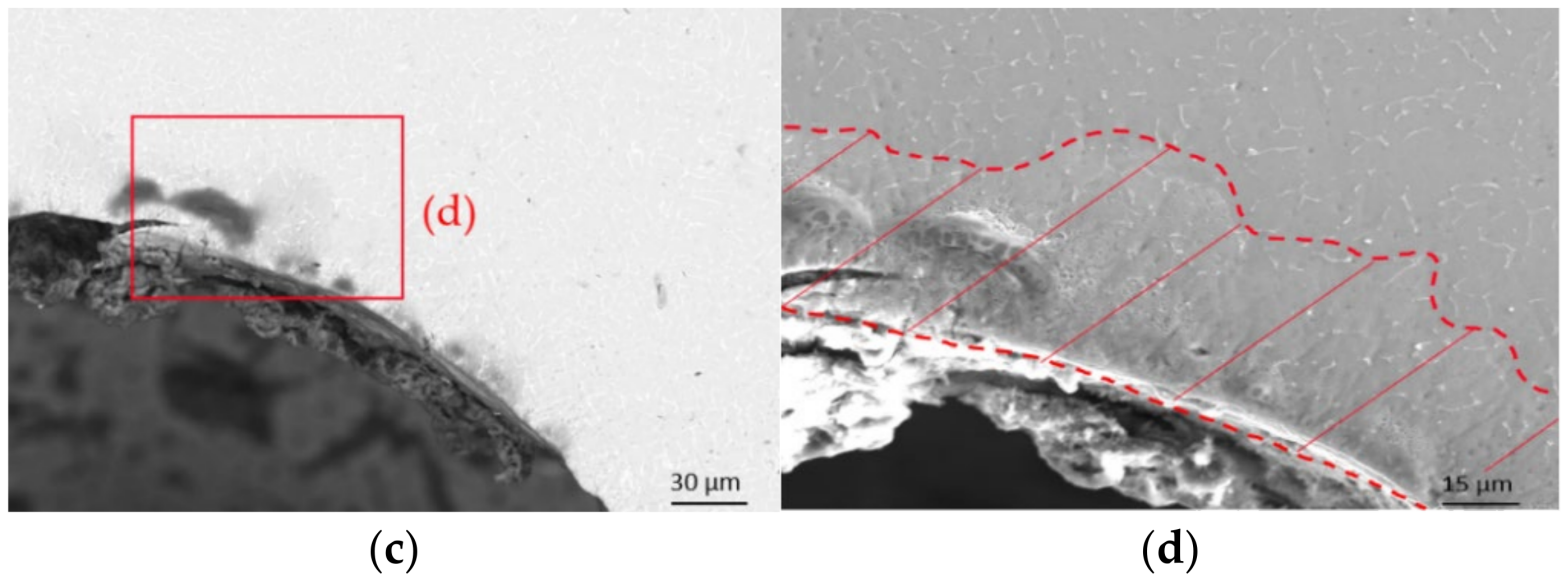
Elemental mapping in the area of a pore of (**a**) aluminum and (**b**) magnesium; (**c**) BSE image of pore edge; (**d**) SE image with highlighted foamy Mg-rich region.

Very similar results were acquired around a large pore with a diameter of ≈600 µm which is shown in Figure 8. The integrated average compositions of the highlighted areas in Figure 7 and Figure 8 revealed almost identical values for Al, Si, Mg, Fe and Mn. However, it is visible in Figure 8a,b that Al is depleted and Mg strongly enriched at the edge of the pore. The darker areas on the BSE image (indicated in Figure 8c) correspond well with the Mg-enrichment visible in Figure 8b. Further zooming into this area utilizing a SE image (Figure 8d), the Mg enriched zones display a foamy microstructure.

## 4. Discussion

It was shown that beam oscillation has a significant effect on the pore formation in the weld of AW6082. However, it is difficult to describe the exact relationship between beam figures and pore formation. Thus, no difference in the structure of the welds was detected, which would indicate a mechanism. The structures of the welds show a wide range of equiaxed grains, which were also detected by Whang et al. [12]. They show the influence of different oscillation directions on grain growth using laser welding. Thus, the circular motion in their study showed a wide range of equiaxed grains. Since the used figures are based on superimposed oscillations with which the circle is also parameterized, a wide range of equiaxed grains was also detected in this study. 

It is evident that the resulting energy fields can promote the outgassing of bubbles. Similar results regarding beam deflection and porosity were also shown by Kabasakaloglu et al. [13] and Chen et al. [14] in their investigation of laser welding, although they only considered one figure (“8” & “∞”). However, by varying different welding parameters, such as focus and frequency, they also presented different modes of energy input.

In this study, it was found that a partial shift of the maximum energy input towards the center of the weld had a positive effect on reducing the porosity, as in the case of series C. Minor improvement regarding pore size and amounts were obtained by using a two-bath technique (series E) in comparison to the reference figure. Surprisingly, series A and B, which should act as a link between a one and two bath technique, gave contradictory results in terms of pore size and number. Since A and B were performed using the same figure type, it is clear that the energy distribution significantly influences the porosity, because the figures have different functions in the energy distribution and are also different in size. 

Using a narrower figure with the intention to produce a narrower weld in order to provide less space for pores to be formed did not prove to be effective. This finding is contradictory to the ones reported by Chen et al. [14]: in their study, the narrowest amplitude (0.7 mm) also had the lowest porosity (14%). However, the narrowest amplitude from Chen et al. [14] was 1.5 larger than the standard amplitude of 0.45 mm used in this study. In the aforementioned studies, welds with different parameters and beam oscillations were investigated, but this was limited to one weld per parameter set, which was never longer than 200 mm. These experiments are comparable to the first set of experiments in this study, where welds with 220 mm were also examined with regard to porosity. In the second set of experiments, it could be shown that both low and high porosity (series D) can be welded reproducibly. 

Kabasakaloglu et al. [13] and Chen et al. [14] showed that frequency affects both porosity and seam geometry. Both studies showed that with higher frequency, the ratio of weld-width to -depth became less. Both concluded that there is no stable keyhole and that it is heat conduction welding. The same conclusion is drawn from this study—the weld is very wide concerning its depth, so it is also assumed to be heat conduction welding. This fact excludes the formation of pores by a keyhole. Likewise, other pore formation mechanisms, such as the penetration of hydrogen into the melt or the formation of a gas phase between aluminum oxide and liquid aluminum, as described by Fujii et al. [7], can be ruled out as the main pore formation mechanism in our study. By mechanical removal of the oxide layer and subsequent airtight packaging, re-oxidation of the aluminum was largely suppressed. A short time window between unpacking and evacuation of the welding chamber kept the progress of oxidation to a minimum. The low oxidation and the vacuum in the chamber also minimized the contributed hydrogen, which is often seen as the main reason for pore formation in other welding processes, as described by Ardika et al. [4].

Zhan et al. [5] and Zhou et al. [6] looked specifically at the outgassing of Mg in AA6061/AA5052 in their studies on EBW and fiber laser welding. The experiments in both studies were performed with rotationally symmetrical heat sources on thicker sheets (3 and 4 mm, respectively), which resulted in wider welds. Thus, in both works, the Mg content was detected concerning the weld depth and the weld center. However, the averaged decreases of Mg were similar to the results of the present work. However, the gradient shown by both Zhan et al. [5] and Zhou et al. [6] was not detected in this work. 

In our study, we demonstrated that the main mechanism of pore formation in the AW6082 alloy under the given parameters (especially vacuum pressure) is the evaporation of magnesium. Zinc plays a minor role since it is present in lower concentrations (~ 0.1 wt.%) compared to magnesium (~0.9 wt.%). A direct correlation between the outgassing of these elements and the beam pattern could not yet be recognized, since at this point of the research only the reference figure R could be investigated with EPMA. Complete avoidance of porosity, therefore, seems unlikely, and further research on this topic is required.

## 5. Conclusions

In this study electron beam welding with newly developed welding configurations was performed on AW6082 plates. The following points comprise the most important findings:

- The beam figure and consequently the energy input influences the porosity, but the direct relationship between beam deflection and pore formation is still unclear.
- It was possible to reproducibly avoid (Series B) and create (Series D) pores.
- The pore size will also be influenced by the beam figure. However, a direct correlation between pore number and pore size could not be established.
- Outgassing of the alloying elements magnesium and zinc were identified as the main pore formation mechanism.
- Considering that magnesium and other elements will never completely outgas from the material, pore-free welding in a high vacuum is improbable.

## Figures and Tables

**Table 1:** Limits of the chemical composition of the aluminum alloy AW6082 [16]. Copyright 2020 Ansys Granata Edupack.

Al	Cr	Cu	Fe	Mg	Mn	Si	Ti	Zn	Others
95.2	0	0	0	0.6	0.4	0.7	0	0	0
98.3	0.25	0.1	0.5	1.2	1	1.3	0.1	0.2	0.15

**Table 2:** Welding figures and their parameters for the second series of experiments. The energy distribution was symmetrical across the welding direction. All figures contained 1000 dots.

Series	Figure Welding Direction →	Energy Distribution	Length [mm]	Width [mm]
A	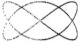	quadratic	1.35	0.45
B	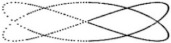	linear	3	0.45
C	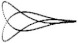	centered	1.35	0.45
D	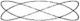	constant	1.35	0.25
E	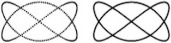	Right Figure 75% Left Figure 25%	3	0.45

**Table 3:** EPMA results from the single spot analyses and the integrated mapping areas; SD: standard deviation; bld.: below detection limit.

Quant Results	Single Spot Analyses	Al	Si	Mg	Fe	Mn	Cr	Cu	Zn	Total
	[n]	[wt.%]	[wt.%]	[wt.%]	[wt.%]	[wt.%]	[wt.%]	[wt.%]	[wt.%]	[wt.%]
Avg. base material	18	96.21	1.06	0.85	0.42	0.64	0.13	0.10	0.13	99.54
±(SD)		0.99	0.30	0.16	0.42	0.24	0.04	0.02	0.02	0.30
Avg. weld	27	96.08	1.27	0.49	0.49	0.68	0.14	0.10	bld.	99.24
±(SD)		0.50	0.28	0.05	0.09	0.04	0.01	0.02	-	0.,29
**Semi-Quant Results**	**Integrated Mapping Area**	**Al**	**Si**	**Mg**	**Fe**	**Mn**	**Cr**	**Cu**	**Zn**	**Total**
	**[mm^2^]**	**[wt.%]**	**[wt.%]**	**[wt.%]**	**[wt.%]**	**[wt.%]**	**[wt.%]**	**[wt.%]**	**[wt.%]**	**[wt.%]**
Avg. base material	38.62 (Figure 7)	96.45	1.12	0.93	0.55	0.56	not analyzed			99.61
Avg. weld	30.19 (Figure 7)	97.02	1.21	0.56	0.63	0.58	not analyzed			99.99
Avg. weld	0.44 (Figure 8 and Figure A1)	96.82	1.18	0.52	0.61	0.58	not analyzed			99.71

## Data Availability

Not applicable.

## Appendix A

**Table A1:** Single spot EPMA analyses of the Al-phase in the base material and in welded areas. bld.: below detection limit.

Single Spot Analysis	Al	Si	Mg	Fe	Mn	Cr	Cu	Zn	Total
	[wt.%]	[wt.%]	[wt.%]	[wt.%]	[wt.%]	[wt.%]	[wt.%]	[wt.%]	[wt.%]
Base material 01	96.74	0.86	0.84	0.06	0.49	0.12	0.08	0.14	99.32
Base material 02	94.47	1.53	0.78	1.35	1.19	0.19	0.12	0.14	99.77
Base material 03	97.16	0.87	0.84	0.16	0.45	0.08	0.12	0.13	99.82
Base material 04	95.56	1.16	0.81	0.67	0.92	0.20	0.07	0.15	99.55
Base material 05	97.44	0.68	0.83	bld.	0.27	0.07	0.08	0.10	99.47
Base material 06	96.47	1.01	0.85	0.38	0.56	0.10	0.10	0.11	99.58
Base material 07	97.02	0.84	0.84	0.11	0.49	0.11	0.09	0.11	99.61
Base material 08	97.23	0.76	0.83	0.05	0.44	0.16	0.07	0.15	99.69
Base material 09	94.57	1.85	1.50	0.99	0.96	0.18	0.12	0.13	100.30
Base material 10	97.02	0.82	0.81	0.09	0.49	0.12	0.10	0.10	99.55
Base material 11	96.80	0.96	0.82	0.11	0.53	0.14	0.14	0.12	99.62
Base material 12	94.62	1.43	0.80	0.90	0.81	0.11	0.10	0.16	98.93
Base material 13	96.83	0.96	0.79	0.16	0.47	0.12	0.14	0.11	99.60
Base material 14	96.99	0.86	0.81	0.13	0.44	0.10	0.09	0.14	99.56
Base material 15	95.74	1.04	0.80	0.48	0.69	0.14	0.06	0.13	99.08
Base material 16	95.06	1.31	0.79	1.16	1.01	0.17	0.11	0.15	99.76
Base material 17	96.69	0.91	0.80	0.19	0.48	0.10	0.10	0.10	99.39
Base material 18	95.31	1.16	0.80	0.64	0.80	0.16	0.08	0.13	99.08
Weld 01	96.02	1.01	0.44	0.58	0.73	0.15	0.12	bld.	99.12
Weld 02	96.08	1.28	0.56	0.50	0.71	0.13	0.12	0.09	99.47
Weld 03	96.45	1.03	0.48	0.49	0.73	0.13	0.08	bld.	99.43
Weld 04	96.29	1.07	0.47	0.43	0.66	0.13	0.11	bld.	99.22
Weld 05	96.51	1.10	0.49	0.36	0.62	0.13	0.11	bld.	99.34
Weld 06	96.28	1.24	0.45	0.44	0.67	0.17	0.08	bld.	99.39
Weld 07	96.30	1.22	0.51	0.39	0.63	0.13	0.15	0.09	99.42
Weld 08	96.43	0.95	0.45	0.55	0.71	0.13	0.11	bld.	99.38
Weld 09	97.40	0.70	0.38	0.27	0.58	0.12	0.09	bld.	99.59
Weld 10	96.36	1.04	0.43	0.57	0.75	0.16	0.08	bld.	99.46
Weld 11	95.68	1.44	0.48	0.57	0.74	0.17	0.12	bld.	99.26
Weld 12	96.02	1.56	0.50	0.54	0.71	0.14	0.09	bld.	99.57
Weld 13	96.83	1.14	0.49	0.51	0.68	0.14	0.10	bld.	99.92
Weld 14	95.46	1.53	0.46	0.69	0.76	0.14	0.11	bld.	99.19
Weld 15	96.60	1.13	0.52	0.42	0.69	0.14	0.10	bld.	99.61
Weld 16	96.42	1.34	0.56	0.47	0.64	0.16	0.07	bld.	99.71
Weld 17	95.76	1.43	0.52	0.54	0.67	0.15	0.11	bld.	99.24
Weld 18	95.67	1.57	0.52	0.51	0.68	0.14	0.09	0.07	99.24
Weld 19	95.71	1.91	0.53	0.52	0.68	0.17	0.13	bld.	99.67
Weld 20	95.69	1.10	0.48	0.52	0.69	0.11	0.07	bld.	98.73
Weld 21	96.04	1.07	0.44	0.52	0.68	0.14	0.13	0.08	99.10
Weld 22	95.34	1.60	0.49	0.45	0.68	0.16	0.10	bld.	98.83
Weld 23	96.07	1.29	0.48	0.47	0.65	0.13	0.11	bld.	99.21
Weld 24	96.01	1.30	0.54	0.44	0.67	0.13	0.09	bld.	99.25
Weld 25	96.34	0.99	0.39	0.27	0.59	0.15	0.06	bld.	98.80
Weld 26	94.98	1.95	0.49	0.57	0.72	0.14	0.11	0.08	99.03
Weld 27	95.32	1.29	0.59	0.50	0.70	0.15	0.12	0.10	98.76
